# The Expression of Three Negative Co-Stimulatory B7 Family Molecules in Small Cell Lung Cancer and Their Effect on Prognosis

**DOI:** 10.3389/fonc.2021.600238

**Published:** 2021-04-15

**Authors:** Meng-jun Qiu, Qin Xia, Yao-bing Chen, Xie-fan Fang, Qiu-ting Li, Li-sheng Zhu, Xin Jiang, Zhi-fan Xiong, Sheng-li Yang

**Affiliations:** ^1^ Division of Gastroenterology, Liyuan Hospital, Tongji Medical College, Huazhong University of Science and Technology, Wuhan, China; ^2^ Institute of Pathology, Union Hospital, Tongji Medical College, Huazhong University of Science and Technology, Wuhan, China; ^3^ Institute of Pathology, Tongji Hospital, Tongji Medical College, Huazhong University of Science and Technology, Wuhan, China; ^4^ Department of Toxicology, Charles River Laboratories, Inc., Reno, NV, United States; ^5^ Cancer Center, Union Hospital, Tongji Medical College, Huazhong University of Science and Technology, Wuhan, China

**Keywords:** small cell lung cancer, immune checkpoint, PD-L1, B7-H3, B7-H4, immunity therapy

## Abstract

**Background:**

In recent years, immune checkpoint inhibitors have shown significant effects in a variety of solid tumors. However, due to the low incidence of small cell lung cancer (SCLC) and its unclear mechanism, immune checkpoints in SCLC have not been fully studied.

**Methods:**

We evaluated the expression of PD-L1, B7-H3, and B7-H4 in 115 SCLC tissue specimens using immunohistochemistry. The clinical data of patients with SCLC were retrospectively reviewed to investigate three negative co-stimulatory B7 family molecules’ ability to affect the prognosis of SCLC.

**Results:**

Among the SCLC patients with complete follow-up data (n = 107), sixty-nine (64.49%) expressed moderate to high B7-H3 levels, which correlated positively with tumor sizes (*P* < 0.001). Eighty (74.77%) patients expressed moderate to high B7-H4 levels, which correlated positively with metastases (*P* = 0.049). The positive expression of B7-H3 and B7-H4 correlated significantly with shortened overall survival (OS) (B7-H3, *P* = 0.006; B7-H4, *P* = 0.019). PD-L1 was positively expressed only in 13.08% of cancer tissues, and there was no significant correlation with prognosis. The Cox proportional hazards regression showed that B7-H3 was an independent prognostic indicator of OS (*P* = 0.028; HR = 2.125 [95% CI = 0.985-4.462]).

**Conclusions:**

Our results suggest that B7-H3 has a negative predictive effect on SCLC. This outcome provides a theoretical basis for the subsequent research on immune checkpoint inhibitors targeting B7-H3.

## Introduction

With changes in the living environment and lifestyle, malignant tumors have become the leading cause of death for most people in the world (1). Among tumors, lung cancer is the most commonly diagnosed cancer and the leading cause of cancer death. Small cell lung cancer (SCLC) accounts for about 15% of lung cancers, has a high degree of malignancy, and is highly invasive (2). Although early SCLC is relatively sensitive to chemoradiotherapy, only a small proportion of patients achieve complete remission. Most patients are prone to drug resistance and relapse, and the treatment is poorly effective on patients in the later stages (3). The mechanism of the occurrence and progression of SCLC is complicated, and knowledge on the corresponding driving genes is lacking. In addition, due to the long-term invasion of SCLC by tobacco carcinogens, its genome is susceptible to complex changes, such as mutations, insertions, deletions, copy number changes, and chromosomal rearrangements, which result in high immunogenicity and high mutation load of SCLC (4–6).

Normally, the body’s immune system plays a role in identifying and clearing tumor cells. While protecting the host from pathogens and cancer, the immune system also plays a vital role in maintaining autoimmune tolerance. The activation of T lymphocytes is the core of the immune response. To induce T cell activation, proliferation and differentiation require dual signal stimulation. First, T cell receptors (TCR) specifically bind to antigen peptide-major histocompatibility complex (MHC) complexes on the surface of antigen presenting cells (APCs) to provide the first signal; Second, the co-stimulatory CD28 receptor on T cells binds to the ligands (B7) expressed on APC to form a B7-CD28 complex, which plays a costimulatory effect. When T cells are activated, CTL antigen 4 receptors are induced to express and compete with CD28 to bind to ligands, preventing excessive T cell activation (7–10). Tumor microenvironments usually contain increased regulatory immunosuppressive T cells, and tumor cells express key negative regulators, such as programmed death ligand-1 (PD-L1) and B7-H3, which inhibit the immune regulatory response induced by T cells, down-regulate or prevent the body’s anti-immune effect, and promote the disabling of immune function to help tumor escape (11, 12). In recent years, tumor immunotherapy has been recognized as the most promising method for curing malignant tumors. The molecular mechanism of tumor immune escape is one of the core issues in tumor immune research. The abnormal expression of molecules of the B7 family in the tumor microenvironment leads to anti-tumor immune suppression and immune escape. The B7 family consists of 10 members, namely B7-1, B7-2, B7-H1 (PD-L1), B7-DC, B7-H2, B7-H3, B7-H4, B7-H5, B7-H6, and B7-H7 (13, 14). In recent years, the role of the B7 family in tumor immunity has received great attention, especially the three molecules of B7-H1, B7-H3, and B7-H4 have become hot research objects. To study their respective roles in the tumor microenvironment, to further block their corresponding signaling pathways is a new approach for tumor immunotherapy. The abnormal expression of B7-H1, B7-H3 and B7-H4 in tumor tissues and tumor microenvironment, as well as the negative regulation of tumor immune response, suggest that these three molecules not only have hidden theoretical research for tumor immunotherapy, it also has potential clinical application value and trans-epochal significance. More and more immune checkpoint inhibitors are being applied in the treatment of SCLC. Nivolumab and Pembrolizumab are humanized IgG4 anti-PD-1 monoclonal antibodies that prevent anti-tumor immune response by binding to the PD-1 receptor on the surface of T cells; they have had significant results in early clinical trials in a variety of tumors (15, 16). Once immunotherapy is effective, its long-term benefit rate to patients may increase significantly. Therefore, screening the dominant population, that is, the exploration of immune markers, is urgent. In this study, three negative co-stimulatory B7 family molecules PD-L1, B7-H3, and B7-H4 were used as research objects to explore their expression and relationship with prognosis in SCLC. The outcome of this research will provide a reliable scientific basis for clinical diagnosis.

## Materials and Methods

### Patient and Clinicopathological Data

The pathological specimens of 120 SCLC patients diagnosed by surgical resection or biopsy at Tongji Hospital, Tongji Medical College, Huazhong University of Science and Technology from January 2012 to August 2017 were collected. The pathological specimens of 5 patients were lost, and, additionally, 8 patients were lost to follow-up. Therefore, we analyzed 107 patients with complete pathological data and follow-up data. Ethical approval was requested and obtained from the Medical Ethics Committee of Tongji Medical College of Huazhong University of Science and Technology. Written informed consent was obtained from all participants.

### Tissue Microarray Construction

Tissue microarrays (TMAs) were prepared using a standard tissue microarrayer (Azumaya Corp., Tokyo, Japan). Avoiding the necrotic area, a 1.1 mm^2^ tumor tissue was obtained from the central area of each tumor, and the area 5 cm beside the tumor was regarded as normal tissue and was used as a control. The TMA section thickness was 4 microns.

### Immunohistochemistry and Evaluation

**Figure 1:**
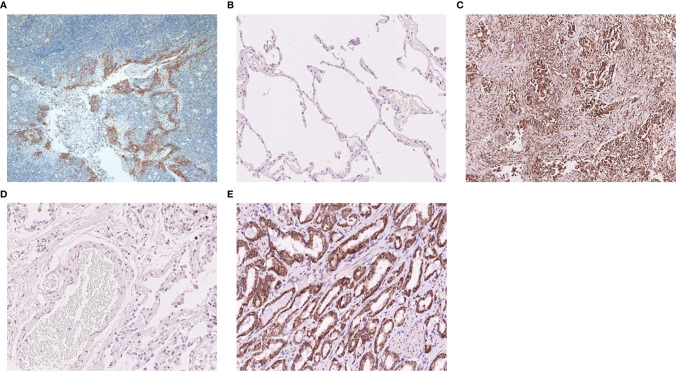
Typical immunohistochemistry of PD-L1, B7-H3, and B7-H4 protein. **(A)** The negative and positive expression of the PD-L1 protein in tonsil tissue. **(B)** The negative expression of the B7-H3 protein in normal lung tissue. **(C)** The positive expression of the B7-H3 protein in adenocarcinoma of the lung. **(D)** The negative expression of the B7-H4 protein in normal lung tissue. **(E)** The positive expression of the B7-H4 protein in prostatic carcinoma.

HE staining and immunohistochemical staining of TMA sections: two clone numbers of PD-L1 were used (clone SP142, dilution 1:50, Ventana and clone 22C3, dilution 1:50, Dako). Staining was achieved with a fully automatic staining instrument and detection system for SP142 and 22C3 were paired Ventana and Dako secondary antibody systems (17–20). The tonsil was used as the control tissue. As shown in **Figure 1A**, the PD-L1 staining characterized of tonsil tissue respectively were negative in superficial squamous epithelium and interfollicular area (as a negative control), and positive in lymphocyte, macrophages, and crypt epithelial cells in the germinal center (as a positive control). As new immune checkpoint, B7-H3 and B7-H4 have a good potential clinical value. At present, B7-H3 and B7-H4 don’t have a wide range of applications in clinical, so the immunohistochemistry and evaluation of these two antibodies don’t have fixed standardized dyeing process and quality control. B7-H3 and B7-H4 have fewer expressions in normal tissues, usually negative (**Figures 1B, D**). We detected normal lung tissues and a variety of cancer tissue microarray, we found that only lung adenocarcinoma and partial prostate cancer significantly high expression (**Figures 1C, E**). B7-H3 (clone D9M2L, dilution 1:100) and B7-H4 (clone D1M8I, dilution 1:100) were purchased from Cell Signaling. Immunohistochemical stainings were evaluated by two independent pathologists unaware of the patients’ clinical results per the following criteria: (1) PD-L1 (SP142): The percentage of staining of positive tumor-infiltrating immune cells (IC) in the entire tumor region (IC, including lymphocytes, macrophages, dendritic cells, and granulocytes). The tumor region was the proportion of tumor cells and their associated intratumoral and peritumoral stroma. The IC staining pattern was dark brown dots or lines. A percentage of < 1% was considered negative, and ≥ 1% was considered positive. (2) PD-L1 (22C3): The percentage of partial or complete membrane stained tumor cells in all living tumor cells present in the sample. A percentage of < 1% was considered negative, and ≥ 1% was considered positive. (3) Positive B7-H3 and B7-H4 were evaluated according to the intensity of the epithelial tumor cell-specific cytoplasmic/membranous staining (0 - negative; 1 - very weak; 2 - moderate; and 3 - strong expression). Staining scores of 0 and 1 were defined as negative staining, whereas 2 and 3 were regarded as positive staining (21).

### Statistical Analysis

Fisher’s exact test was used to analyze the relationship between PD-L1, B7-H3, and B7-H4 protein expressions and clinicopathological parameters. OS was defined as the date of death or the date of the last follow-up. The survival curve was drawn using the Kaplan-Meier method, and the *P* value comparison was performed using the log-rank test. We used Cox proportional hazards models to assess the significance of B7-H3 and B7-H4 in univariate and multivariate analyses. A *P* value of < 0.05 was considered statistically significant. All analyses were performed using the SPSS 21 software package (IBM, Armonk, NY).

## Results

### Clinical Pathological Data

The immunohistochemical staining of PD-L1, B7-H3, and B7-H4 for the TMAs of 115 small cell lung cancer patients and their adjacent tissues was performed, of which 1 pair of the immunohistochemical images were absent from B7-H3, and 3 pairs were absent from B7-H4. All patients were followed up to September 6, 2019, but 8 were lost to follow-up. The median time of the patients was 18 months (1-57 months). The clinicopathology of the 107 cases of SCLC evaluated is shown in **Table 1**. The study included 92 men (85.98%) and 15 women (14.02%). The median age was 59 years (40 to 79 years). The majority of patients underwent chemotherapy (81.31%), a few (12.15%) underwent surgery. Seventy-three patients (68.22%) had lymph node metastasis, and 39 patients (36.45%) had distant metastasis.

**Table 1:** Clinicopathological characteristics of patient samples and the expression of PD-L1, B7-H3, and B7-H4 in small cell lung cancer.

Characteristics	Number of cases (%)
Age (years)	
<50	23 (21.50)
≥50	84 (78.50)
Gender	
Female	15 (14.02)
Male	92 (85.98)
Tumor size	
<5 cm	52 (48.60)
≥5cm	54 (50.47)
NA	1 (0.93)
Lymph node status	
N 0	34 (31.78)
N 1-3	73 (68.22)
Metastasis	
No	68 (63.55)
Yes	39 (36.45)
Operation	
No	94 (87.85)
Yes	13 (12.15)
Radiotherapy	
No	67 (62.62)
Yes	37 (34.58)
NA	3 (2.80)
Chemotherapy No Yes NA	13 (12.15) 87 (81.31) 7 (6.54)
Expression of B7-H3	
Negative	37 (34.58)
Positive	69 (64.49)
NA	1 (0.93)
Expression of B7-H4	
Negative	24 (22.43)
Positive	80 (74.77)
NA	3 (2.80)
Expression of PD-L1	
Negative	93 (86.92)
Positive	14 (13.08)

NA, not available.

**Figure 2:**
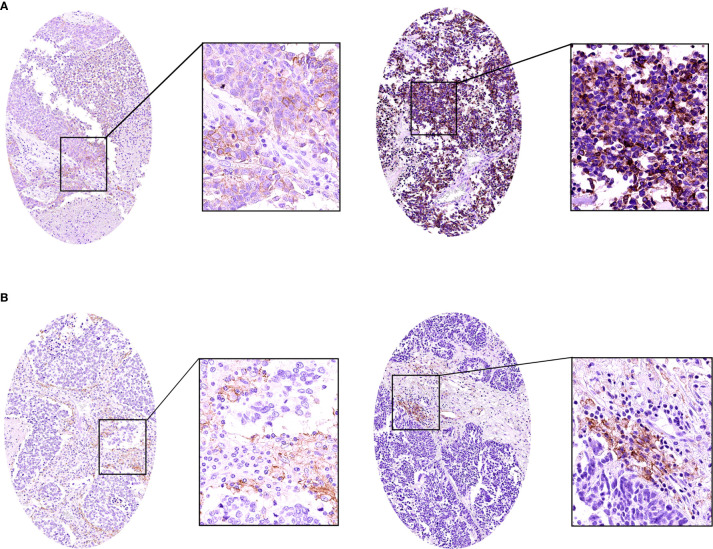
The immunohistochemistry of the PD-L1 protein in small cell lung cancer (SCLC). **(A)** The positive expression of the PD-L1 protein in SCLC. **(B)** The positive expression of the PD-L1 protein in SCLC tumor-infiltrating immune cells.

**Figure 3:**
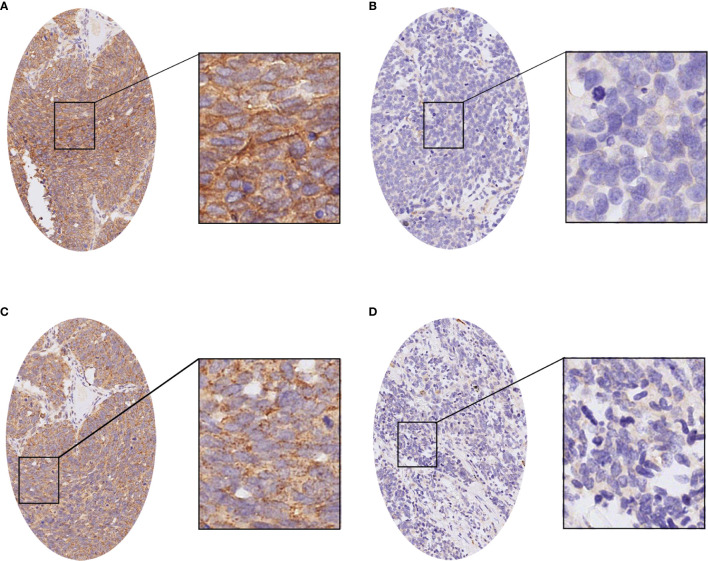
The immunohistochemistry of the B7-H3 and B7-H4 proteins in small cell lung cancer (SCLC). **(A)** The positive expression of the B7-H3 protein in SCLC. **(B)** The negative expression of the B7-H3 protein in SCLC. **(C)** The positive expression of the B7-H4 protein in SCLC. **(D)** The negative expression of the B7-H4 protein in SCLC.

We analyzed the expression of PD-L1 in tumor cells and tumor-infiltrating immune cells. The positive expression of PD-L1 in tumor cells was presented as partial or complete staining of the cell membrane (**Figure 2A**). The positive expression of PD-L1 in tumor-infiltrating immune cells was presented as dark brown dots or lines (**Figure 2B**). The positive expression of B7-H3 and B7-H4 in TMA membranes or cytoplasms (or both) showed different degrees of yellow-brown staining (**Figures 3A, C**). There were also negative expressions of B7-H3 and B7-H4 (**Figures 3B, D**). The positive expression rates of PD-L1, B7-H3, and B7-H4 were 13.08%, 64.49%, and 74.77%, respectively. As shown in **Table 2**, the B7-H3 protein expression was significantly related to tumor size (*P* < 0.001), and the B7-H4 protein expression was significantly related to distant metastasis (*P* = 0.049). However, the association between the PD-L1 protein expression and clinicopathological characteristics was not statistically significant.

**Table 2:** Correlation between the expressions of PD-L1, B7-H3, and B7-H4 and clinicopathologic characteristics of small cell lung cancer patients.

Variables	PD-L1		*p*-values^a^	B7-H3		*p*-values^a^	B7-H4		*p*-values^a^
	Negative	Positive		Negative	Positive		Negative	Positive	
Age (years) <50 ≥50	19 74	4 10	0.494	7 30	16 53	0.805	6 18	16 64	0.580
Gender Female Male	13 80	2 12	1.000	3 34	12 57	0.250	3 21	12 68	1.000
Tumor size <5 cm ≥5cm NA	47 45 1	5 9 0	0.471	27 10 0	25 43 1	<0.001*	16 8 0	36 43 1	0.158
Lymph node status N 0 N 1-3	29 64	5 9	0.763	10 27	23 46	0.660	10 14	24 56	0.325
Metastasis No Yes	58 35	10 4	0.568	28 9	40 29	0.090	20 4	48 32	0.049*
Operation No Yes	80 13	14 0	0.209	31 6	62 7	0.370	20 4	71 9	0.491
Radiation No Yes NA	56 34 3	11 3 0	0.587	22 13 2	45 23 1	0.465	18 5 1	46 32 2	0.164
Chemotherapy No Yes NA	13 73 7	0 14 0	0.211	5 29 3	8 57 4	0.791	2 20 2	11 64 5	0.745

a Fisher’s exact test

^*^indicates p < 0.05.

### Clinical Results

**Figure 4:**
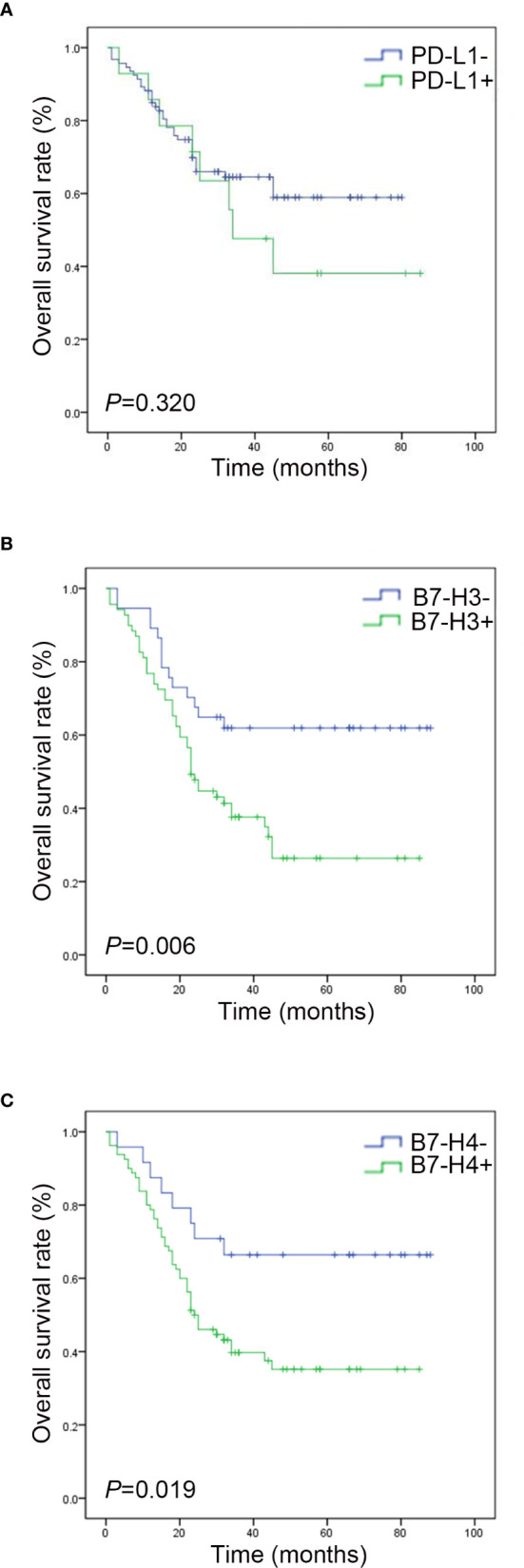
Survival analysis of PD-L1, B7-H3, and B7-H4 expressions in small cell lung cancer. **(A)** The survival curve shows that the total survival rate of the PD-L1 negative group is higher than that of the PD-L1 positive group (*P* > 0. 05). **(B)** The survival curve shows that the total survival rate of the B7-H3 negative group is higher than that of the B7-H3 positive group (*P* < 0. 05). **(C)** The survival curve shows that the total survival rate of the B7-H4 negative group is higher than that of the B7-H4 positive group (*P* < 0. 05).

The Kaplan Meier survival curve analysis showed no significant correlation between PD-L1 expression and survival time (**Figure 4A**). However, compared with the negative expression group (B7-H3, 23.81 ± 10.968 months; B7-H4, 28.42 ± 18.050 months), patients with positive B7-H3 or B7-H4 expression had shorter OS times (B7-H3, 7.39 ± 3.965; B7-H4, 8.61 ± 3.987 months) (B7-H3, *P* = 0.006; B7-H4, *P* = 0.019) (**Figures 4B, C**). In the univariate analysis, the clinical and pathological features associated with shorter OS were men (*P* = 0.048), tumor size ≥ 5 cm (*P* = 0.008), lymph node metastases (*P* = 0.005), distant metastases (*P* < 0.001), without chemotherapy (*P* = 0.003), B7-H3 positive expression (*P* = 0.008), and B7-H4 positive expression (*P* = 0.024) (**Table 3**). Gender, tumor size, lymph node metastasis, distant metastasis, chemotherapy, B7-H3 expression, and B7-H4 expression were included in the multivariate analysis of Cox proportional hazard models. The results showed that the B7-H4 protein expression was not an independent prognostic factor of OS (Hazard ratio = 1.789; 95% confidence interval (CI): 0.933-3.256; *P* = 0.251). In contrast, the negative expression of B7-H3 in small cell lung cancer was an independent prognostic factor that improves OS (Hazard ratio = 2.125; 95% CI: 0.985-4.462; *P* = 0.028) (**Table 4**).

**Table 3:** Univariate analyses of various prognostic parameters in patients with small cell lung cancer cox-regression analysis.

	Univariable analysis		
Variables	Hazard Ratio	95% CI	*p*
Age (< 50 VS ≥ 50)	1.064	0.585-1.936	0.840
Gender (female VS male)	2.521	1.008-6.306	0.048*
Tumor size (< 5 cm VS ≥ 5cm)	2.057	1.204-3.513	0.008*
Lymph node status (N0 VS N1-3)	2.469	1.308-4.662	0.005*
Metastasis (No VS Yes)	2.727	1.638-4.539	<0.001*
Operation (Yes VS No)	1.334	0.632-2.254	0.087
Radiation (Yes VS No)	2.346	1.233-4.721	0.105
Chemotherapy (Yes VS No)	2.936	1.178-5.236	0.003*
PD-L1 (negative VS positive)	1.321	0.563-2.578	0.603
B7-H3 (negative VS positive)	2.251	1.233-4.110	0.008*
B7-H4 (negative VS positive)	2.371	1.120-5.018	0.024*

CI, confidence interval.

^*^indicates p < 0.05.

**Table 4:** Multivariate analyses of various prognostic parameters in patients with small cell lung cancer cox-regression analysis.

Variables	Multivariable analysis					
	Hazard Ratio	B7-H3		Hazard Ratio	B7-H4	
		95% CI	*p*		95% CI	*p*
Gender (female VS male)	3.237	1.360-8.141	0.067	3.066	1.128-8.943	0.087
Tumor size (< 5 cm VS ≥ 5cm)	1.326	0.722-2.157	0.811	1.432	0.668-2.544	0.293
Lymph node status (N0 VS N1-3)	2.133	1.051-3.739	0.042*	2.167	0.985-3.968	0.068
Metastasis (No VS Yes)	2.514	1.369-5.322	0.012*	2.437	1.346-4.378	0.016*
Chemotherapy (Yes VS No)	3.261	1.546-7.445	0.023*	3.238	1.435-7.521	0.025*
B7-H3 (negative VS positive)	2.125	0.985-4.462	0.028*	–	–	–
B7-H4 (negative VS positive)	–	–	–	1.789	0.933-3.256	0.251

CI, confidence interval. ^*^indicates p < 0.05.

## Discussion

The immune system is an important regulatory system in tumor biology that develops and coordinates its attack mode, through its judgment of self and non-self, and restores an equilibrium state. Theoretically, host T cells can distinguish tumor cell antigens and destroy tumors, but, in reality, there are very few tumor cells cleared by the immune system. This is because cancer cells can escape the immune system through a variety of ways and use this metamorphic “self” protection method to proliferate and spread in the body (22).

The occurrence and development of SCLC are hidden and complicated processes. Also, continuous or intermittent chemoradiation stimulation can easily lead to the emergence of new SCLC antigens released from apoptotic tumor cells, which makes traditional treatment methods unable to effectively identify tumor cells and exert their anti-cancer effect (23). Through the in-depth study of tumor microenvironments, an important class of immune checkpoint molecules – the negative co-stimulatory B7 family molecules – has been uncovered and has attracted more and more attention in recent times. As inhibitory ligands, after the negative co-stimulatory B7 family molecules bind to their receptors, they regulate T cell proliferation and cytokine secretion negatively at a certain threshold of T cell receptor attack, damage T cell immune function in tumor microenvironments, and induce the inactivation of infiltrating T cells, enabling tumor cells to escape immune surveillance, maintain peripheral tolerance, and promote tumor development. At the same time, the secretion of many cytokines, including IL-10 and IFN-γ, is also affected (24).

In the SCLC tissue samples, we found that B7-H3 and B7-H4 were highly expressed positively. The expressions of these molecules on cancer cells were dispersed in the whole section locally or sporadically and correlated with larger tumor sizes and the tendency to metastasize. The expression of both molecules was associated with shorter survival time. However, the expression of PD-L1 had nothing to do with clinicopathological features and OS. Additionally, we found that the expression of PD-L1, B7-H3, and B7-H4 in adjacent tissues was significantly lighter than that in the corresponding cancer tissues, indicating that the three molecules did change in SCLC.

B7-H3 expression is reportedly associated with a decrease in T cell proliferation and interferon-γ production, and B7-H3 blockade can lead to an increase in CD8+T cell influx and antitumor effects (25, 26). There is evidence that B7-H4 can be used as a co-inhibitor of T cell responses (27, 28). The overexpression of B7-H4 promotes the proliferation of FOXP3+ regulatory T cells and the secretion of IL-10 and TGF-β1, inhibiting antigen presentation cell function and exerting immune tolerance in tumor microenvironments (29–31).

Furthermore, we performed a Spearman correlation analysis based on the intensity scores of the expression of B7-H3 and B7-H4 and found that the two correlated significantly (the Spearman correlation was 0.533, *P* < 0.001). We believe that some common mechanisms promote the overactivation of inhibitory signals and biological activities in tumor cells. Yes, this study only revealed the expression of three negative co-stimulatory B7 family molecules in SCLC and their correlation with prognosis. Therefore, the specific molecular pathway mechanism needs further research.

Admittedly, our research has certain limitations. During the TMA production process, the quality of the staining of some images was poor, and there was an over- or under-labeling of target proteins due to the methods and materials used. Our results also contradict findings from other studies. In our study, the positive rate of PD-L1 was only 13.08%, whereas, this rate is highly variable (0-71.6%) in SCLC in other evaluations (32, 33). The differences in this rate may stem from many different factors during the expression of the negative co-stimulatory B7 family molecules, such as the choice of cancer species, the quality of the sample, and the sensitivity to reagents. All of the above factors may have significant effects on the expression of PD-L1, B7-H3, and B7-H4. As a subtype of neuroendocrine cancer, although the treatment of small cell lung cancer was mainly radiotherapy and could achieve good therapeutic effects, there were still some patients asked for surgical resection of the lesion, so our data also had higher part of patients undergoing the operation. In our research, PD-L1, B7-H3, and B7-H4 were not related to surgery, radiotherapy and chemotherapy. Our study was a retrospective study. Most patients had no record of immune check point inhibitors therapy, and chemoradiotherapy was the main treatment for SCLC. Therefore, the expression levels of these three negative co-stimulatory B7 family molecules were of little significance to the treatment modalities and we could not know among all those treatment modalities which affect the most on immune check point inhibitors expression. In the future, this will also be the direction we need to further study.

Targeted immunotherapy refers to a therapeutic method that acts on the immune system, blocks the immunosuppressive signal, and produces an effective anti-tumor response by targeting immune cells. Enoblituzumab (MGA271, Macrogenics) is an optimized monoclonal antibody for B7-H3 targeted therapy. As of October 12, 2018, there were 133 patients with melanoma expressing B7-H3. Head and neck squamous cell carcinoma (SCCHN), non-small cell lung cancer (NSCLC), and urothelial patients have been evaluated in phase I clinical studies for the significant therapeutic effect of MGA271 alone or in combination with anti-PD-1 monoclonal antibodies (34). Drug development and trials for B7-H4 are currently underway (35). In SCLC, further understanding the regulation of B7-H3 and B7-H4 expression, the recognition of their cognate receptors, and their immunomodulatory role in cancer will be key to supporting further clinical development. Meanwhile, exploring immunosuppressive combination therapy strategies for different targets to supplement the single-drug treatment plan is very effective in the omission of other targets and delayed response.

The detection of the expression of negative co-stimulatory B7 family molecules in patients’ tumor tissues can help to clinically evaluate the efficacy of checkpoint antibody drugs and the selection of patients. Closely monitoring immune checkpoints during disease diagnosis and treatment can further guide the targeting and programing of tumor immunotherapy. The joint development of immunological checkpoint therapeutic monoclonal antibodies and accompanying diagnostic reagents is an inevitable trend for targeted immune checkpoint therapy in the future. In order to develop more efficient combined immunization programs, we must constantly explore new therapeutic targets and biomarkers. Therefore, we need to pay attention to the specificity of tumor area microenvironments and explore the key immune checkpoint molecules involved in the different stages of cancer evolution. We hope to use advanced diagnostic technology and integrate multidisciplinary resources to jointly study the processes of tumor imbalance and immune escape.

## Conclusions

In summary, we analyzed the expression of three different negative co-stimulatory B7 family molecules in human SCLC using immunohistochemistry and clinical data. Surgery was an inappropriate treatment for most SCLC patients, while chemotherapy and radiotherapy were the better choices. The main chemotherapy regimens were etoposide and cisplatin (56%), followed by irinotecan and cisplatin (30%), and 81.31% of patients received chemotherapy. Surgery and radiotherapy had no significant effect on the prognosis of SCLC patients, but chemotherapy could significantly improve the survival time of patients. However, PD-L1, B7-H3, and B7-H4 were not related to surgery, radiotherapy, and chemotherapy. Both B7-H3 and B7-H4 suppress the immune system, although the impact of B7-H3 is more significant.

## Data Availability Statement

The raw data are available upon request on the following e-mail address: d201981842@hust.edu.cn.

## Ethics Statement

The studies involving human participants were reviewed and approved by the Medical Ethics Committee of Tongji Medical College of Huazhong University of Science and Technology. The patients/participants provided their written informed consent to participate in this study. Written informed consent was obtained from the individual(s) for the publication of any potentially identifiable images or data included in this article.

## Author Contributions

S-LY and Z-FX conceived and designed the experiments. M-JQ and QX carried out the research. Y-BC, X-FF, Q-TL, and L-SZ contributed the analysis tools and analyzed the data for the study. M-JQ wrote the paper. QX and XJ revised the manuscript. All authors contributed to the article and approved the submitted version.

## Funding

This study was supported by the National Key Research and Development Program of China (2018YFC2002000), and the National Natural Science Foundation of China (81874232).

## Conflict of Interest

XF was employed by Charles River Laboratories, Inc.

The remaining authors declare that the research was conducted in the absence of any commercial or financial relationships that could be construed as a potential conflict of interest.
